# Identification of Novel Clinically Relevant Variants in 70 Southern Chinese patients with Thoracic Aortic Aneurysm and Dissection by Next-generation Sequencing

**DOI:** 10.1038/s41598-017-09785-y

**Published:** 2017-08-30

**Authors:** Miaoxian Fang, Changjiang Yu, Siyao Chen, Weiping Xiong, Xin Li, Rong Zeng, Jian Zhuang, Ruixin Fan

**Affiliations:** 1 Department of Intensive Care Unit of Cardiac Surgery, Guangdong Cardiovascular Institute, Guangdong General Hospital, Guangdong Academy of Medical Sciences, Guangdong, China; 2 Department of Cardiac Surgery, Guangdong Cardiovascular Institute, Guangdong General Hospital, Guangdong Academy of Medical Sciences, Guangdong, China

## Abstract

Thoracic Aortic Aneurysm and Dissection (TAAD) is a life-threatening pathology and remains challenging worldwide. Up to 40% of TAAD are hereditary with complex heterogeneous genetic backgrounds. Recently, next-generation sequencing (NGS) has been successfully applied to identify genetic variants in an efficient and cost-effective manner. In our study, NGS coupled with DNA target-capture array was used to screen 11 known causative genes of TAAD in 70 patients from Southern China. All the identified variants were confirmed by Sanger sequencing. We identified forty variants in 36 patients (51.4%), including three known pathogenic (7.5%), 10 likely pathogenic variants (25%, 9 in *FBN1*, 1 in *ACTA2*), and 27 variants with uncertain significance (VUS) (67.5%). Among the 27 VUS, 14 (51.9%) were in the *FBN1* gene, 3 in *Col5A2*, 2 in *ACTA2*, 2 in *MYH11*, 2 in *MYLK*, 2 in *SLC2A10*, 1 in *MSTN* and 1 in *SMAD3* respectively. Based on the segregation data and independent reports, five known likely pathogenic variants and four VUS were upgraded to pathogenic variant and likely pathogenic variant respectively. Our data indicate that NGS is a highly efficient genetic method for identification of pathogenic variants in TAAD patients.

## Introduction

Thoracic aortic aneurysm (TAA) is life-threatening disease with significant morbidity and mortality. The actual incidence of TAA is hard to estimate due to the fact that it can be non-symptomatic until the onset of acute aortic dissection (AD) or rupture. Thoracic aortic aneurysm and dissection (TAAD) may develop into a highly lethal situation. The incidence of acute AD has been reported to be approximately 2–3.5/100,000 inhabitants per year in the US^1^. It is estimated that individual lifetime risk of rupture or dissection reaches 34% by the time aorta achieves a diameter of 6 cm^2^. Since TAAD at the early stage is silent and non-symptomatic, early detection and evaluation of the risk of onset and disease progression are crucial for avoiding life-threatening situations.

Previous studies have described the complex and heterogeneous genetic background of TAAD which is often associated with connective tissue diseases including Marfan syndrome (MFS), Ehlers-Danlos syndrome (EDS), Shprintzen-Goldberg syndrome (SGS) and Loeys–Dietz syndrome (LDS). The term symptomatic TAAD is used for characterizing this special group of TAAD patients^3^. However, it was reported that TAAD patients with family history took up approximately 20–40% of the TAAD population^4^. Since identification of the very first mutation in the *FBN1* in MFS^5, 6^, causative mutations have subsequently been identified in a dozen others including *COL3A1* and *COL5A2* mutations associated with EDS pathogenesis^7, 8^, *TGFBR2* mutations associated with Marfan-like syndrome^9^, and *TGFBR1*, *TGFBR2* and *SMAD3* associated with LDS^10–12^. Gene mutations including *ACTA2*, *MSTN*, *MYH11*, *MYLK*, and *SLC2A10* ^8, 11, 13–17^ are associated with vascular diseases and are responsible for TAAD.

The high throughput, multiplexing next-generation sequencing (NGS) is currently regarded as the most powerful technology for genetic testing in clinical settings. NGS application as a screening method for the diagnosis of TAAD has been reported in Caucasian population. Proost *et al*. screened 14 genes from 55 patients and identified 15 pathogenic mutation and six variants of uncertain significance (VUS)^18^; Ziganshin *et al*. recruited a group of 102 patients with 21 genes examined and found that 4% of the patients had likely pathogenic variants and 22% had VUS^19^; In a cohort of 175 patients, Wooderchak-Donahue *et al*. found 51 rare variants in 10 selected genes^20^, in which pathogenic variants presented in 10% patients and VUS in 18% patients.

In the current study, 11 TAAD-associated genes including *ACTA2*, *Col3A1*, *Col5A2*, *FBN1*, *MSTN*, *MYH11*, *MYLK*, *SLC2A10*, *SMAD3*, *TGFBR1*, and *TGFBR2* were analyzed by NGS among 70 TAAD subjects enrolled from southern China. Forty variants were identified in 36 TAAD patients. Among all the variants, 12 pathogenic/likely pathogenic variants were in *FBN1* gene, one likely pathogenic variant was in *ACTA2* gene, and the other 27 VUS presented in eight genes.

## Results

### Clinical findings

The clinical characteristics of TAAD patients were summarized in Table 1. The mean age at the time of genetic testing was 45.7 years. Age of the patients ranged from 18 to 66 years. Sixty (85.7%) patients were male and 10 (14.3%) patients were female. Thirteen (18.6%) patients had a family history of TAAD. Twenty-four (34.3%) patients revealed a history of tobacco use, while 11 (15.7%) patients revealed a history of alcohol use.

**Table 1:** Clinical characteristics of the study group.

Clinical characteristics of the study group, n = 70	
Male (n,%)	60(85.7)
Female (n,%)	10(14.3)
Age(mean, SD)	45.7(8.5)
Tobacco use (n,%)	24(34.3)
Alcohol use (n,%)	11(15.7)
Family history (n,%)	13(18.6)
Suspected MFS (n,%)	15(21.4)
CAD (n,%)	5(7.1)
Hypertension (n,%)	31(44.3)
Hyperlipidemia (n,%)	18(25.7)
*Associated structural abnormalities*	
Thoracic AD (n,%)	43(61.4)
TAA (n,%)	27(38.6)
BAV (n,%)	4(5.7)
Other CV*	28(40.0)

AD: aortic dissection, BAV: Bicuspid aortic valve, CAD: coronary artery disease, CV: cardiovascular, Other CV*: includes findings such as mitral valve prolapse, tricuspid valve prolapse, mitral regurgitation or congenital heart disease, thick aortic valve, TAA: thoracic aortic aneurysm.

A variety of structural abnormalities associated with TAAD were identified among several patients: 43(61.4%) patients had thoracic AD, TAA was present in 27 (38.6%) patients. Bicuspid aortic valve (BAV) was present in 4 (5.7%) patients. Twenty-eight (40%) patients had other cardiovascular diseases. Five (7.1%) patients had coronary artery disease (CAD). Thirty-one (44.3%) patients had hypertension. Eighteen (25.7%) patients were hyperlipidemic. Fifteen (21.4%) patients were suspected of MFS, with their clinical features and identified variants shown in Table 2. Eight (53.3%) had a positive family history, all included patients have aortic root dilatation z score ≥2, eight included patients have systemic score >=7 points. Cardiovascular, skeletal, ectopia lentis, dural, skin and lung problems were identified in 15(100%), 15(100%), 9(60%), 1(6.7%), 1(6.7%) and 3(20.0%) of patients, respectively. Therefore, there are 12 patients diagnosed MFS based on clinical data, and 3 (TAAD003, TAAD024, TAAD038) based on genetic examinations according to the revised Ghent criteria.

**Table 2:** Clinical Data and Variants identified in suspected Marfan syndrome patients.

Family	Cardiova-scular	Skeletal	ectopia lentis	Dural ectasia	skin	lung	Z score	System-ic score	Family History (Y/N)	Fulfill Ghent Criteria before genetic test (Y/N)	Gene	Classification
TAAD003	+	+	−	−	−	−	12.91	5	N	N	*FBN1*	Likely patho
TAAD005	+	+	+	−	−	−	11.11	≥7	N	Y	*FBN1*	VUS
TAAD013	+	+	+	−	−	−	8.87	≥7	N	Y	*FBN1*	Likely patho
TAAD020	+	+	−	−	+	−	24.44	≥7	Y	Y	*FBN1*	VUS
TAAD023	+	+	−	−	−	+	22.06	≥7	N	Y	*FBN1*	VUS
TAAD024	+	+	−	−	−	+	18.11	4	N	N	*FBN1*	Patho
TAAD036	+	+	+	−	−	−	15.83	≥7	Y	Y	*FBN1*	VUS
TAAD038	+	+	−	−	−	−	4.92	5	N	N	*FBN1*	Likely patho
TAAD039	+	+	+	+	−	−	4.47	≥7	Y	Y	*FBN1*	VUS
TAAD045	+	+	−	−	−	−	14.19	4	Y	Y	*FBN1*	Likely patho
TAAD046	+	+	+	−	−	+	4.01	≥7	Y	Y	*FBN1*	VUS
TAAD056	+	+	+	−	−	−	8.54	≥7	N	Y	*FBN1*	Patho
TAAD068	+	+	+	−	−	−	9.48	6	Y	Y	*FBN1*	Likely patho
TAAD069	+	+	+	−	−	−	6.16	5	Y	Y	*FBN1*	Likely patho
TAAD070	+	+	+	−	−	−	7.02	6	Y	Y	*FBN1*	VUS

+:positive, −;negative; Patho: pathogenic; VUS: variants of uncertain significance.

### Mutation analysis

In a total of 70 analyzed TAAD patients, 40 rare variants were identified in 36 patients (36/70, 51.4%) (Tables 2 and 3). Twenty-seven of these 40 identified rare variants (67.5%) were novel findings. Variants were classified in line with recommendations from the American College of Medical Genetics (ACMG)^21^ based on the following information: (I) published data including functional and clinical information, (II) variant frequency in the dbSNP and Exome Variant Server and presence in any public variant databases, (III) conservation of the altered residue, (IV) computational prediction programs for variant causality including splicing effects: SIFT^22^ and PolyPhen^23^, and (V) family segregation studies. Available evidences for each new variant were evaluated by two independent reviewers.

**Table 3:** Variants identified in *FBN1* genes.

Family	Disease	Family History(Y/N)	Nucleotide	Protein	Type	Classification	Reference
TAAD001	AD + AR	N	c.7204+6T > G	—	Splicing	VUS	*
TAAD003	MFS + AD + AR	N	c.1098G > T	p.Trp366Cys	Missense	Likely patho	25
TAAD005	MFS + AD + AR	N	c.8123A > G	p.Asn2708Ser	Missense	VUS	44
TAAD011	TAA + AR	N	c.1129T > A	p.Cys377Ser	Missense	VUS	*
TAAD013	MFS + TAA + AR	N	c.7471 delA	p.Thr2491ProfsX191	Frameshift	Likely patho	*
TAAD014	TAA + AR	N	c.1496G > C	p.Cys499Ser	Missense	Likely patho	27
TAAD017	AD	N	c.7754T > C	p.Ile2585Thr	Missense	patho	45
TAAD020	MFS + TAA + AR	Y	c.4049G > A	p.Cys1350Tyr	Missense	VUS	46
TAAD021	TAA + AR	Y	c.5084G > T	p.Cys1695Phe	Missense	VUS	*
TAAD023	MFS + TAA + AR	N	c.7988G > A	p.Cys2663Tyr	Missense	VUS	*
TAAD024	MFS + AD	N	c.8147_8148insA	p.Tyr2716Ter	Frameshift	patho	18
TAAD026	AD	N	c.911G > T	p.Cys304Phe	Missense	VUS	*
TAAD030	AD	N	c.5742C > A	p.Cys1914Ter	Nonsense	Likely patho	*
TAAD034	AD	N	c.8069T > G	p.Met2690Arg	Missense	VUS	*
TAAD036	MFS + MR + TR	Y	c.5579G > T	p.Cys1860Phe	Missense	VUS	46
TAAD038	MFS + AD + AR	N	c.4454G > A	p.Cys1485Tyr	Missense	Likely patho	28
TAAD039	MFS + AD + AR	N	c.2627G > T	p.Cys876Phe	Missense	VUS	*
TAAD045	MFS + TAA + AR	Y	EX5_54 DEL	—	Exon deletion	Likely patho	*
TAAD046	MFS + AD + AR	Y	c.2216G > A	p.Cys739Tyr	Missense	VUS	*
TAAD050	TAA + AR + MR	Y	EX4_53 DEL	—	Exon deletion	Likely patho	*
TAAD051	AD	Y	c.2056G > A	p.Ala686Thr	Missense	VUS	29
TAAD053	AD	Y	c.7567A > C	p.Ile2523Leu	Missense	VUS	*
TAAD056	MFS + AR + MR	N	c.247 + 1G > A	—	Splicing	patho	47
TAAD068	MFS + TAA + AR	Y	c.4292G > A	p.Cys1431Tyr	Missense	Likely patho	32
TAAD069	MFS + AR + MR	Y	c.8292_8293insT	p.Phe2764PhefsX10	Frameshift	Likely patho	*
TAAD070	MFS + TAA + AR	Y	c.6801C > A	p.Asn2267Lys	Missense	VUS	18

*This study.

The underline patients were with concomitant mutations in different genes.

AD: aortic dissection; AR: aortic regurgitation; MFS: Marfan syndrome; MR: mitral regurgitation; TAA: thoracic aortic aneurysm; TR: tricuspid regurgitation; Patho: pathogenic; VUS: variants of uncertain significance.

**Figure 1:**
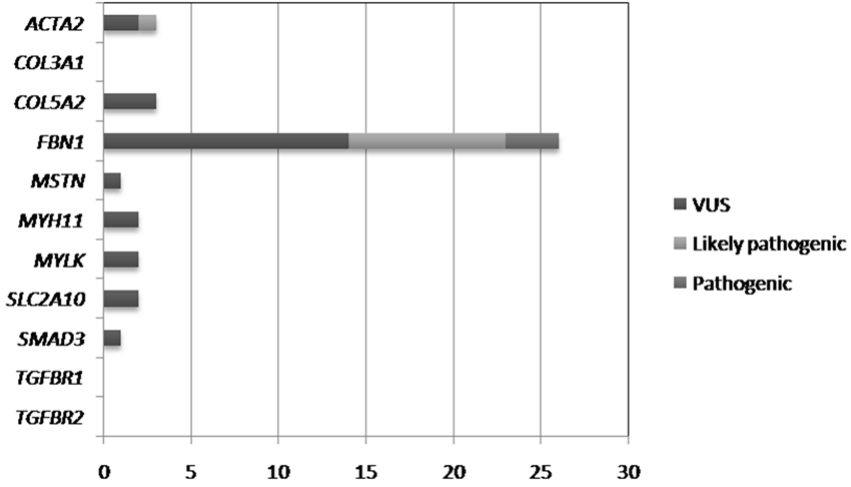
Showed the distribution of identified variants in TAAD associated genes in the current study. Among all the 40 variants, 3 were pathogenic (all in *FBN1* gene), 10 were likely pathogenic (9 for the *FBN1* gene, 1 for the *ACTA2* gene), and 27 were VUS (14 in *FBN1* gene, 3 in *Col5A2*, 2 in *ACTA2*, 2 in *MYH11*,2 in *MYLK*, 2 in *SLC2A10*,1 in *MSTN* and 1 in *SMAD3* respectively).

Based on software estimations, functional analysis and the segregation data, it was assumed that among all the 40 variants, 3 were pathogenic (7.5%, all in *FBN1* gene), 10 were likely pathogenic (25.0%, 9 for the *FBN1* gene, 1 for the *ACTA2* gene), and 27 were VUS (67.5%). Among the 27 VUS, 14 (51.8%) were in the *FBN1* gene, 3 in *Col5A2*, 2 in *ACTA2*, 2 in *MYH11*, 2 in *MYLK*, 2 in *SLC2A10*, 1 in *MSTN* and 1 in *SMAD3* respectively. The distribution of identified variants in TAAD associated genes were shown in Fig. 1.

#### FBN1

Similar to results reported by other studies^18, 20, 24^, the present study again confirmed that most of the variants were found in *FBN1* gene, which is the primary causative gene in TAAD. As listed in Table 3, three pathogenic mutations, c.7754T > C missense mutation (TAAD017), c.8147_8148insA frameshift mutation (TAAD024), and c.247 + 1G > A splicing mutation (TAAD056) were identified. In addition, we detected four reported likely pathogenic mutation, including c.1098G > T (TAAD003), c.1496G > C (TAAD014), c.4454G > A (TAAD038) and c4292G > A (TAAD068). According to the latest ACMG recommendations for interpreting and reporting sequence variations^21^, these four likely pathogenic variants could be reclassified into pathogenic variants due to previous independent reports^25–32^. Five out of these seven probands were diagnosed as MFS, and four of them were with family history.

Importantly, five novel variants, including 2 frameshift (c.7471delAand c.8292_8293insT), 2 exon deletion (EX5_54 DEL and EX4_53 DEL)and a missense mutation (c.5742C > A) in *FBN1* gene were classified as likely pathogenic variants. Mutation c.7471delA in case TAAD013 without family history located in the Von Willebrand factor type A (vWA) domain, which is a hallmark of blood coagulation protein von Willebrand Factor. Truncation of the vWA domain may affect function of protein Fibrillin 1^33^. Another frameshift mutation c.8292_8293insT identified in case TAAD069 with MFS is located in the Asprosin domain and was carried by the patient’s sister without any symptom yet, whose long term follow-up was scheduled. The EX5_54 DEL mutation was identified in a 52-year-old female with MFS (TAAD045). A family history of the disease was found in her father (42 y) and son (16 y), both of whom died due to rupturing of dissecting aneurysm developed from MFS. The second Exon deletion mutation EX4_53 DEL (TAAD050) was found in a 21-year male patient diagnosed with TAA as well as severe aortic valve and mitral valve regurgitation. Family history revealed that his mother died of AD at the age of 38.The remaining novel nonsense mutation c.5742C > A was detected in a 41-year-old male with Stanford A AD (TAAD030) located in the calcium-binding epidermal growth factor (ebEGF) modules, contributing to early termination of encoded protein for the amino acid, producing truncated protein and eventually causing great changes on the structure and function of the protein.

Additionally, fourteen VUS in *FBN1* were found. Seven out of them have confirmed TAAD family history (TAAD020, TAAD021, TAAD036, TAAD046, TAAD051, TAAD053, TAAD070). Nine were reported for the first time while the other 5 had been reported by other studies. A large majority of them were missense mutation (13/14, 92.9%). Notably, two novel VUS, c.5084G > T, p.Cys1695Phe (TAAD021) and c.7988G > A, p.Cys2663Tyr (TAAD023), were similar to pathogenic mutations of c.5084G > A, p.Cys1695Tyr^26^ and c.8121G > C, p.Cys2663Ser^27^ described in previous studies in terms of mutation site. Aside from that, SIFT and PolyPhen protein function had both been proved to be detrimental. Thus, these variants were highly likely to be pathogenic. Although 4 novel VUS for *FBN1*, c.1129T > A (TAAD011), c.911G > T (TAAD026), c.2627G > T(TAAD039) and c.2216G > A (TAAD046) were predicted to be detrimental by SIFT and PolyPhen protein function prediction, their pathogenicity is still not well established.

#### ACTA2

A previously reported missense mutation variant c.635G > A (p.Arg212Gln) in the ACTIN domain of *ACTA2* ^34–36^ was carried by a 48-year-old male (TAAD027) with diagnosis of Stanford A AD, ascending aortic aneurysm, and severe aortic valve insufficiency in the current study. This mutation was originally classified as likely pathogenic and elevated to pathogenic with regard to the fact that it was proved by two dependent studies and predicted harmful by SIFT and PolyPhen software. No family history was found in this case, and family verification were negative.

In addition, 2 novel missense VUS in *ACTA2* (TAAD041, TAAD065) were found in the current study in absence of family history. Their clinical significance and pathogenesis have yet to be elucidated.

### Other TAAD associated genes

Besides 14 VUS found in *FBN1*, 2 VUS in *ACTA2*, as well as 11 other VUS, including 3 in *Col5A2*, 2 in *SLC2A10*, 2 in *MYH11*, 2 in *MYLK*, 1 in *MSTN* and 1 in *SMAD3* were identified in the current study (Table 4). Almost all VUS (9/11, 81.8%) were missense mutation. Detailed information was listed in Table 4.

**Table 4:** Thirteen novel VUS identified in other TAAD associated genes.

Gene	Family	Disease	Family History(Y/N)	Nucleotide	Protein	Type
ACTA2	TAAD041	AD	N	c.401T > C	p.Met134Thr	Missense
ACTA2	TAAD064	AD	N	c.574A > G	p.Met192Val	Missense
COL5A2	TAAD046	MFS + AD + AR	Y	c.2846C > G	p.Ser949Cys	Missense
COL5A2	TAAD054	AD + AR	N	c.3388A > G	p.Lys1130Glu	Missense
COL5A2	TAAD061	AD	N	c.3856G > A	p.Asp1286Asn	Missense
MSTN	TAAD022	AD	N	c.747 + 8A > G	—	Splicing
MYH11	TAAD034	AD	N	c.1523G > A	p.Arg508His	Missense
MYH11	TAAD040	AD	N	c.1907A > G	p.Asp636Gly	Missense
MYLK	TAAD019	AD + AR	N	c.4882G > A	p.Val1628Met	Missense
MYLK	TAAD065	AD	N	c.3923A > G	p.Tyr1308Cys	Missense
SLC2A10	TAAD016	AD + AR	N	c.1154C > T	p.Ala385Val	Missense
SLC2A10	TAAD051	AD	Y	c.1456G > T	p.Ala486Ser	Missense
SMAD3	TAAD051	AD	Y	c.532 + 9G > A	—	Splicing

The underline patients were with concomitant mutations in different genes.

To be noted, more than one VUS in different genes were found in 3 probands. Case TAAD034 with AD had two novel heterozygous variants: c.8069T > G in *FBN1* and c.1523G > A in *MYH11*.Although no family history was confirmed in this patient, c.1523G > A in *MYH11*was predicted to be pathogenic by two programs (SIFT and PolyPhen) while c.8069T > G in *FBN1* showed a nonpathogenic result. Thus, *MYH11*c.1523G > A was more likely than *FBN1*c.8069T > G to be pathogenic. TAAD046 was diagnosed MFS and AD with confirmed family history. His mother died of AD (<40 y), and his two brothers were both diagnosed as MFS. The old brother received Betall operation but died of cerebral hemorrhage. The young brother died due to rupture of AD. This proband had two novel VUS (*FBN1* c.2216G > A and *COL5A2* c.2846C > G), both of which were missense variants. Further prediction on the protein function was performed indicating that *FBN1* c.2216 G > A was harmful both by SIFT and Polyphen while *COL5A2*c.2846C > G was pathogenic by SIFT and non-pathogenic by Polyphen, respectively. However, *COL5A2* c.2846C > G was downgraded to likely benign after familial targeted sequencing revealed that the variant was present in his unaffected grandson. At this point, we believe that *FBN1* c.2216G > A was much likely to be the pathogenic mutation. Case TAAD051 with Stanford A AD had two missense and one splicing mutation (*FBN1* c.2056G > A, *SLC2A10* c.1456G > T and *SMAD3* c.532 + 9G > A). *FBN1* c.2056G > A has been reported in a previous study with unknown clinical significance^29, 37^, while *SLC2A10* c.1456G > T and *SMAD3* c.532 + 9G > A were novel identified variants in this study. All of them were similarly predicted to be pathogenic and non-pathogenic by SIFT and PolyPhen protein function prediction, respectively. Except for the pathology of aortic artery, further investigation of this patient showed no clinical presentation of MFS, EDS or LDSIII such as abnormality of skin, crystalline, skeleton as well as osteoarthritis and so on so forth. Family history also revealed that the patient had an affected father, but his DNA was unavailable. Therefore, the pathogenicity of these mutations remained uncertain.

### Family confirmation

**Figure 2:**
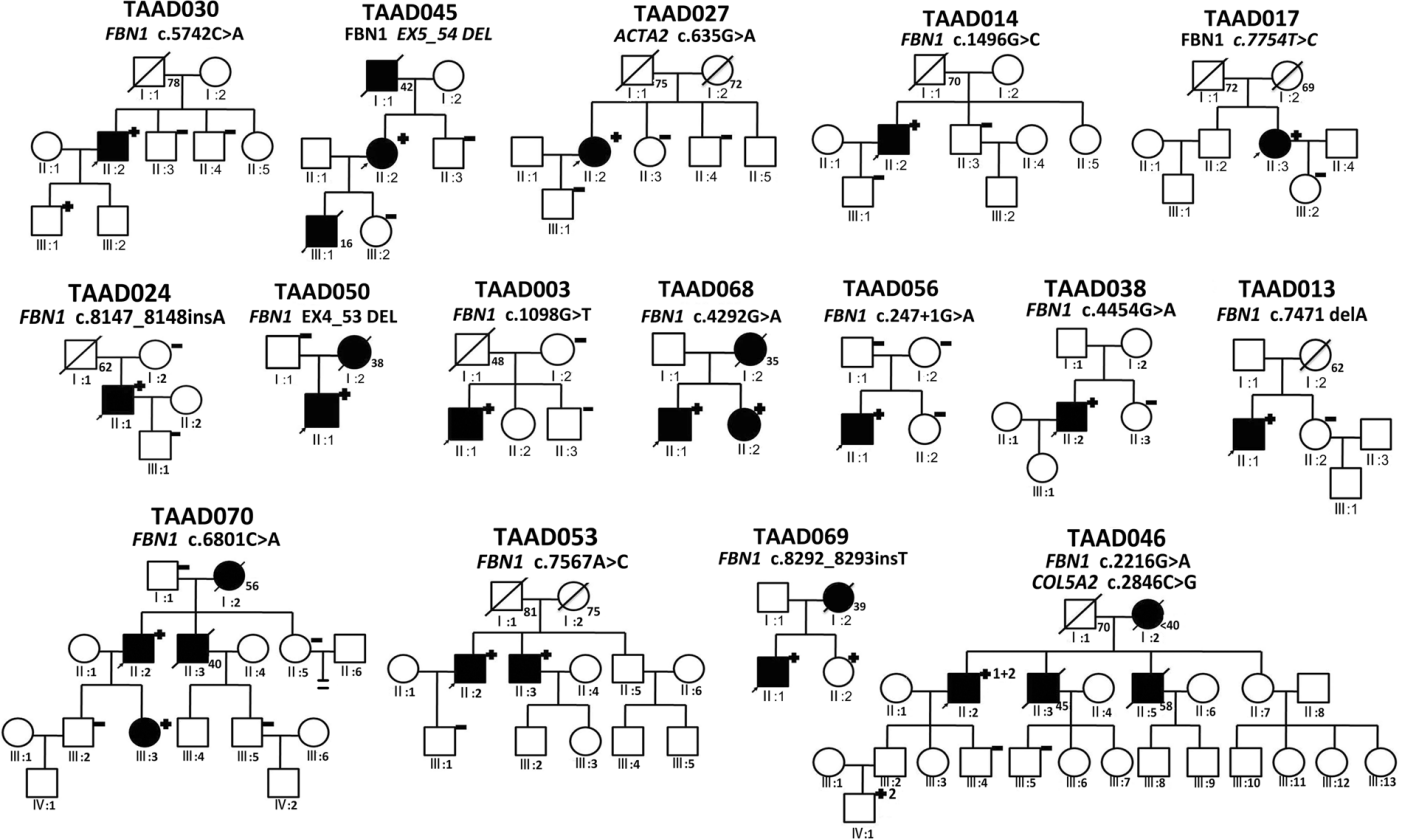
Provided the pedigrees of families with pathogenic and likely pathogenic mutations as well as 3 VUS cases with positive family verifications. Five members from different families were found to have same pathogenic variants with probands (TAAD030, TAAD053, TAAD068, TAAD069, TAAD070).

Furthermore, 21 of 36 probands were further verified in the family, including 13 with pathogenic/likely pathogenic and 8 VUS with family history. Among the 21 verified families, 5 members from different families were found to have same pathogenic variants with probands (TAAD030, TAAD053, TAAD068, TAAD069, TAAD070). Three of them (TAAD053-brother, TAAD068-sister, TAAD070-daugther) had been previously diagnosed with TAAD. Therefore, the pathogenicity of 2 VUS, *FBN1* c.7567A > C in TAAD053 and *FBN1* c.6801C > A in TAAD070 respectively, were validated and upgraded in accordance of ACMG guideline. The other two members (TAAD030-son, TAAD069-sister) were identified as pathogenic mutation carrier in our study. Both of them received imaging test and laboratory examination for TAAD subsequently. Results revealed abnormality of skeleton and crystalline and systemic score ≥ 7 in TAAD030-son meeting the diagnostic criteria of MFS. Detailed clinical characteristics of genotype positive probands and family members were shown in Table 5, pedigrees of these families were shown in Fig. 2.

**Table 5:** Clinical characteristics of probands with pathogenic/likely pathogenic mutation and related mutation carriers.

Family	Position on pedigree-status	Sex	Age	Type Of syndr-ome	CVS involve-ment of the aorta	Aortic root	AR	LV-EF	Extensi-on	Other CVS involve-ment	Type of surgery	Age at surg-ery	Gene	Mutation
TAAD 003	II:1-prob-And	M	28	MFS	AD	56	3	60	R, As, Ar, D, Ab	NO	B + TAR + DASI	28	*FBN1*	c.1098G > T
TAAD 013	II:1-prob-and	M	35	MFS	AD	65	3	45	R, As, Ar, D, Ab	MR(1), TR(1)	B + TAR + DASI	35	*FBN1*	c.7471 delA
TAAD 014	II:2-prob-and	M	42	N	TAA	68	3	44	NO	MR(1), TR(1)	B	42	*FBN1*	c.1496G > C
TAAD 017	II:3-prob-and	F	43	N	AD	42	2	55	As, Ar, D, Ab	MR(1), CAD	B + TAR + DASI + CABG	43	*FBN1*	c.7754T > C
TAAD 024	II:1-prob-and	M	33	MFS	AD	82	3	71	R, As, Ar, D, Ab	TR(1)	B + TAR + DASI	33	*FBN1*	c.8147_8148insA
TAAD 027	II:2-prob-and	M	48	N	AD	67	3	42	R, As,	MR(1), TR(1)	B	48	*ACTA2*	c.635G > A
TAAD 030	II:2-prob-and	M	41	N	AD	49	2	75	Ar, D, Ab	MR(1)	B + TAR + DASI	41	*FBN1*	c.5742C > A
TAAD 030	III:1-son	M	18	MFS	N	32	0	67	NO	NO	NA	NA	*FBN1*	c.5742C > A
TAAD 038	II:2-prob-and	M	25	MFS	AD	90	3	70	As, Ar, D, Ab	MR(1)	B + TAR + DASI	25	*FBN1*	c.4454G > A
TAAD 045	II:2-prob-and	F	52	MFS	TAA	54	2	67	No	MR(1), TR(1)	B	52	*FBN1*	EX5_54 DEL
**TAAD 046**	II:1-prob-and	M	66	MFS	AD	65	2	55	As, Ar, D, Ab	NO	B + TAR + DASI	66	*FBN1*	c.2216G > A
													*COL5A2*	c.2846C > G
**TAAD 046**	IV:1-gran-dson	M	22	N	N	31	0	72	NO	NO	NA	NA	*COL5A2*	c.2846C > G
TAAD 050	II:1-prob-and	M	21	N	TAA	45	0	58	NO	MR(3), TR(1)	B + MVR	21	*FBN1*	EX4_53 DEL
**TAAD 053**	II:2-prob-and	M	55	N	AD	38	2	68	As, Ar, D, Ab	NO	DAV + TAR + DASI	55	*FBN1*	c.7567A > C
**TAAD 053**	II:3-broth-er	M	56	N	AD	Surge-ry	Sur-gery	65	AS	NO	B	51	*FBN1*	c.7567A > C
TAAD 056	II:1-prob-and	M	24	MFS	AD	72	3	61	As, Ar, D, Ab	MR(3), TI(3)	B + TAR + DASI + MVR + TVP	24	*FBN1*	c.247 + 1G > A
TAAD 068	II:1-prob-and	M	18	MFS	TAA	51	2	65	NO	NO	B	18	*FBN1*	c.4292G > A
TAAD 068	II:2-sister	F	21	MFS mutation carrier	N	33	0	60	NO	NO	NA	NA	*FBN1*	c.4292G > A
TAAD 069	II:1-prob-and	M	23	MFS	TAA	48	1	72	NO	MR(3), TR(1)	B + MVR + TVP	23	*FBN1*	c.8292_8293insT
TAAD 069	II:2-sister	F	21	MFS mutate on carrier	N	29	0	68	NO	NO	NA	NA	*FBN1*	c.8292_8293insT
**TAAD 070**	II:2-prob-and	M	45	MFS	TAA	52	3	55	NO	TR(3)	B + TVP	45	*FBN1*	c.6801C > A
**TAAD 070**	III:3-daug-hter	F	20	MFS	TAA	45	2	60	NO	NO	NA	NA	*FBN1*	c.6801C > A

The underline families were with VUS and have positive family history and verification.

Ab: abdominal aorta, AD: aortic dissection, AR: aortic regurgitation(0 none,1 mild, 2 moderate, 3 severe), Ar: aortic arch, As: thoracic ascending aorta, B: Bentall procedure, CABG: coronary artery bypass grafting, CVS: cardiovascular system, D: thoracic descending aorta, DAV: David procedure, DASI: descending aorta stent implantation, F: female, LVEF: left ventricular ejection fraction, M: male, MFS: Marfan syndrome, MR: mitral regurgitation (0 none, 1 mild, 2 moderate, 3 severe), MVR: mitral valve replacement, N: none, NA: not applicable, TAA: thoracic aortic aneurysm, TAR: total aortic arch replacement, TR: tricuspid regurgitation (0 none, 1 mild, 2 moderate, 3 severe), TVP: tricuspid valve plastic.

## Discussion

NGS for molecular diagnosis of TAAD has recently become a practical screening method to identify disease-related gene mutations, which offers the patient and physician an opportunity to intervene and prevent emergency events for patients and their families. In the current study, NGS was performed to determine mutations in 11 candidate gene associated with TAAD in 70 patients from Southern China. Forty variants, were identified in eight genes in 36 TAAD patients (36/70, 51.4%), among whom 66.7% (24/36) patients had novel variants. The total pathogenic/likely pathogenic variants were 13, in which 5 pathogenic were reclassified from likely pathogenic in this study. Only 10 out of these 36 patients (10/36, 27.8%) showed hypertension and 3(3/36, 8.3%) showed hyperlipidemia, which supports the genetic origin of TAAD. There were 13 cases (13/70, 18.6%) with family history in the current study, which approached the range of 20–40% family history as reported previously^5^. When family history was taken into consideration, the mutation detection rate was 92.3% (12/13) compared with 45.6% (26/57) in non-family history cases. This result implies a greater chance of taking genetic tests among TAAD patients with positive family history.

*FBN1* was first documented as an associated gene with MFS^38–40^. More and more studies have shown that patient with a pathogenic *FBN1* mutation is at risk for developing Marfan-like syndromes such as severe cardiovascular, skeletal, and ophthalmologic complications^5, 6, 20^ and *et al*. Faivre L *et al*.^40^ pointed out that exons 24–32 represented a hotspot for neonatal MFS and severe forms of MFS. Some recent researches also indicated that variants of *FBN1* was strongly related to the developing of TAAD^18, 24^ aside from MFS. Similarly, most variants identified in our TAAD patients located in *FBN1* gene, including 12 pathogenic/likely pathogenic variants and 14 VUS. The majority of them were missense mutations. In the 26 patients with *FBN1* mutations, 15 were diagnosed as MFS, the remaining were TAAD. The data imply that *FBN1* is the primary disease-causative gene for TAAD in the population of Southern China.

Besides pathogenic mutation discussed above, there were a total of 27 variants found in the current study, which were considered as VUS, owe to insufficient data from segregation study and ambiguous results by software estimation. Most of them were missense mutation, which lead to the difficulties to verify their pathogenicity. Nevertheless, in accordance with recommendations from ACMG, pathogenicity of 3 novel and 1 reported VUS in *FBN1* were further established through family validation or previous data. Missense mutation c.7567A > C in TAAD053 and c.6801C > A in TAAD070 can be upgraded because the same mutation was detected in their affected family members. The other two novel VUS, c.5084G > T (p.Cys1695Phe) from TAAD021 and c.7988G > A (p.Cys2663Tyr) from TAAD023, were considered to be highly pathogenic due to the fact that they were same at the site and characteristic to the previous-reported pathogenic mutation. Based on that, we believed these 4 VUS could be reclassified to the likely pathogenic category.

Interestingly, three of 70 patients (4.3%) were found to carry more than one VUS in different genes in this study, c.8069T > Gin *FBN1* and c.1523G > A in *MYH11* (TAAD034); c.2216G > A in *FBN1* and c.2846 C > G in *COL5A2* (TAAD046); c.2056G > A in *FBN1*, c.1456G > T in *SLC2A10* and c.532 + G > A in *SMAD3* (TAAD051). Concomitant mutations in different genes in TAAD patients have been reported in previous studies^41^, thus resulting in the complexity and difficulty of discovering the pathogenicity for TAAD. Therefore, family verification on the pathogenicity of these variants is strongly recommended and results must be carefully evaluated and defined by comparing to other independent studies in combination with prediction outcomes of protein function. As in the current study, the pathogenicity of c.2216G > A in *FBN1* was confirmed and c.2846C > G in *COL5A2* was proved to be benign after family verification of the proband TAAD046. Notably, the three patients with concomitant mutation in different genes were both diagnosed as AD and suffered a severe situation. Therefore, it remained yet to be investigated whether concomitant multiple variants in different genes predict disease severity. Further investigation is needed in order to address this question.

Finally, to translate our findings to clinical practice, all patients carrying pathogenic/likely pathogenic variants and with family history of TAAD were verified and further examined by imaging study. Close follow-ups were scheduled for all these patients. Until now, five out of 21 families were with positive validation, and two members (TAAD030-son, TAAD069-sister) from these 5 families had never received clinical examinations and no previous histories of disease reported. Further clinical examination confirmed their diagnosis of TAAD and they were treated accordingly, suggesting that use of NGS might be particularly useful in determining underlying genetic predisposition for TAAD. Efficient molecular findings combined with NGS can be used to guide optimal management, surveillance, and timely treatment in order to alter the natural course of TAAD.

This study had several limitations. Firstly, the sample size was not large enough. Even though most subjects recruited in the present study were typical and comparatively young, often complicated with MFS diagnoses, and with little to no history of hypertension, further study with a large sample is needed to verify the findings in the present study. Secondly, for patients with VUS, family validation was only performed when family history existed, which might lead to omission of some potential positive information. Thirdly, out of the 11 candidate genes, there could be other mutations among the unselected genes, which could hardly be detected due to the limitation of current methodology and technology. A whole exome sequencing by our team is currently being pursued to overcome these shortcomings.

Our findings broaden the spectrum of genetic backgrounds for thoracic aneurysms and dissections, introducing genetic background as a potential prognostic factor for clinical evaluation of patients with TAAD. Our data has established the *FBN1* gene as the most common causative gene in a TAAD patient population from Southern China.

## Materials and Methods

### Patients

The present study was approved by the ethics committee of Guangdong General Hospital, Guangzhou, China. All experiments were performed in accordance with relevant guidelines and regulations. The study cohort included 70 unrelated patients with TAAD hospitalized in the department of cardiac surgery at Guangdong General Hospital from April 2015 to March 2016. The inclusion criteria were age above 18 years old, born and raised in southern China with only southern China family members, diagnosed as TAAD. For the diagnosis of TAAD, the patients meet the following standards according to the AFFC/AHA Guidelines for the diagnosis and treatment of thoracic aortic disease (2010)^1^. (1) True aneurysm and dissection involving the thoracic aorta. (2) Aneurysm (or true aneurysm): a permanent localized dilatation of an artery, having at least a 50% increase in diameter compared to the expected normal diameter of the artery in question. (3) Aortic dissection: disruption of the media layer of the aorta with bleeding within and along the wall of the aorta. Rupture of thoracic aortic artery caused by trauma and pseudoaortic aneurysm were excluded in this study. Age at diagnosis, gender, tobacco use, alcohol use, hypertension history, hyperlipidemia history, the status of the cardiovascular system, history of AD and surgeries were recorded. A family history of TAAD and other diseases was collected. A generation pedigree was drawn for every individual patient and family. Revised Ghent criteria^42^ was used to define MFS for the suspected and a detailed questionnaire was applied to define the involvement of other systems and organs. A Doppler echocardiographic study and CT scan of the entire aorta were performed for all included patients. The presence of mitral valve prolapses (MVP) and mitral regurgitation (MR) was determined using echocardiography and data concerning the mitral valve recorded. Family history was defined as the presence of more than one patient with TAAD in the family. All participants were informed about the study procedures and informed consent for genetic testing and permission to results publication was signed.

### Genetic testing

Genetic testing was performed using NGS coupled with a DNA target-capture array on an IlluminaHiSeq. 2500 platform by BGI (Shenzhen, China) as previously reported^43^. Briefly, eleven genes (*ACTA2*, *Col3A1*, *Col5A2*, *FBN1*, *MSTN*, *MYH11*, *MYLK*, *SLC2A10*, *SMAD3*, *TGFBR1*, and *TGFBR2*) (Table 6) relevant to TAAD were selected for one capture array (NimbleGen, Roche, Madison, WI, USA), which was designed mainly to capture the CDS of 2,181 known pathogenic genes associated with 561 Mendelian diseases based on the GeneReviews (NCBI) and Genetics Home Reference. Genomic DNA from peripheral blood or abortion tissues were fragmented into lengths ranging from 200 bp to 250 bp. The primers, adapters and indexes were then ligated to the DNA fragments to construct libraries. The DNA fragments were pooled and hybridized to the capture array. After hybridization and enrichment, the DNA sample was sequenced on IlluminaHiSeq. 2500 Analyzers to generate paired-end reads (90 bps).

**Table 6:** List of analyzed genes.

Gene	GenBank accession no	Chromosomal locus	Exons	Protein name	Disease	Reference
ACTA2	NM_001613	10q22–q24	9	Smooth muscle actin, alpha 2	TAAD	11
COL3A1	NM_000090	2q31	51	Collagen type III alpha 1	Ehlers–Danlos type IV	7
COL5A2	NM_000393	2q14-q32	55	Collagen type V alpha 2	Ehlers–Danlos syndrome	8
FBN1	NM_000138	15q21.1	66	Fibrillin 1	Marfan/MASS/TAAD	5
MSTN	NM_005259	2q32.2	3	myostatin	myodystrophy	8
MYH11	NM_002474	16p13.13–p13.12	41	Myosin heavy chain 11	TAAD-patent ductus arteriosus	14
MYLK	NM_053025	3q21	34	Myosin light chain kinase	TAAD	16
SLC2A10	NM_030777	20q13.1	5	Solute carrier family 2 (facilitated glucose transporter) member 10	Arterial tortuosity	13
SMAD3	NM_005902	15q22.33	9	Mothers against decapentaplegic homolog (SMAD) 3	Loeys–Dietz syndrome type III	12
TGFBR1	NM_004612	9q33–q34	9	Transforming growth factor, beta receptor 1	Loeys–Dietz syndrome TAAD Marfan type II	10
TGFBR2	NM_003242	3p22	7	Transforming growth factor, beta receptor 2	Loeys–Dietz TAAD Marfan Type II	9

MASS: Mitral valve prolapse, TAAD: Thoracic aortic aneurysm and dissection.

Short reads mapping, alignment were performed using BWA software (Burrows Wheeler Aligner). SNPs and indels were detected using the SOAPsnp software and GATK IndelGenotyper (http://www.broadinstitute.org/gsa/wiki/index.php/, The Genome Analysis Toolkit) respectively. All reference sequences were based on the NCBI37/hg19 assembly of the human genome (a novel mutation of IDS gene in a Chinese patient with mucopolysaccharidosis II by NGS).

Interpretation of all variants referred to the latest version of ACMG standards and guidelines for the interpretation of sequence variants (PMID:25741868). Normal population frequencies information was from dbSNP, HapMap databases, one thousand genome project database, 100 Chinese healthy adults, EXAC database, NHLBI GO Exome Sequencing Project (ESP). Other information was from HGMD database, published literatures, OMIM database, NCBI-books. In silico deleterious effect was evaluated using PolyPhen and SIFT programs.

### Statistical analysis

All results for categorical variables were presented as numbers and percentages and for continuous variables as the mean and standard deviation (SD). Calculations were performed using SPSS v 20.0.
